# Spironolactone Plus Full-Dose ACE Inhibition in Patients with Idiopathic Membranous Nephropathy and Nephrotic Syndrome: Does It Really Work?

**DOI:** 10.3390/ph3010001

**Published:** 2010-01-05

**Authors:** Paolo Cravedi, Varusca Brusegan, Piero Ruggenenti, Ruth Campbell, Giuseppe Remuzzi

**Affiliations:** 1 Clinical Research Center for Rare Diseases “Aldo e Cele Daccò”, Villa Camozzi, Ranica, Italy; 2 Department of Medicine and Transplantation, Ospedali Riuniti-Mario Negri Institute for Pharmacological Research, Bergamo, Italy; 3 Division of Nephrology, Department of Medicine, University of Alabama, Birmingham, Birmingham, AL 35294, USA

**Keywords:** idiopathic membranous nephropathy, spironolactone, proteinuria, hyperkalemia

## Abstract

We have studied the effects of add-on spironolactone treatment (100 mg/day) in 11 patients with idiopathic membranous nephropathy (IMN) and >3 gm proteinuria/day despite angiotensin converting enzyme (ACE) inhibitor therapy titrated to a systolic/diastolic blood pressure <120/80 mmHg. Blood pressure, 24-hour urinary protein excretion, and creatinine clearance were measured prior to, after two months of combined therapy, and after a 2-month withdrawal period of spironolactone. While systolic and diastolic blood pressure decreased significantly after spironolactone therapy, proteinuria did not improve. Serum potassium increased significantly as well, with three patients requiring resin-binding therapy. Thus, spironolactone seems to have no additional antiproteinuric effects over ACE inhibitor therapy in patients with IMN and nephrotic syndrome and carries the risk of significant hyperkalemia.

## 1. Introduction

There is increasing evidence that aldosterone may play a role in mediating the effects of angiotensin II. Aldosterone can exert a direct nephrotoxic effect through a series of mechanisms, which go beyond a pure mineralocorticoid property and include hemodynamic effects and sympathetic activation. Moreover, this hormone induces fibroblast proliferation and vascular remodelling through the promotion of cytokine and growth factor release, such as transforming growth factor-β [1,2]. In the remnant kidney model, infusion of aldosterone blunted the beneficial effect of angiotensin II receptor blockade on proteinuria, hypertension and glomerulosclerosis [3]. Conversely, adrenalectomy limited hypertension, proteinuria and structural renal injury in rats with subtotal nephrectomy [4]. Blockade of the mineralocorticoid receptor by spironolactone increased the antiproteinuric effect of ACE inhibition in experimental malignant nephrosclerosis [5]. More importantly, spironolactone has been reported to further decrease proteinuria in patients with chronic kidney disease already on chronic treatment with ACE inhibitors suggesting that aldosterone antagonists may also add to the renoprotective effect of RAAS inhibition in humans [2,6,7,8].

Thus, combined antialdosterone and ACE inhibitor therapy may offer new avenues of treatment, in particular for CKD with nephrotic range proteinuria unresponsive to ACE inhibitor alone who are at highest risk of progression to end stage renal disease (ESRD). This prompted us to evaluate the antiproteinuric effect of spironolactone in patients with nephrotic syndrome secondary to idiopathic membranous nephropathy (IMN) and persistent proteinuria despite full dose ACE inhibitor therapy.

## 2. Results and Discussion

### 2.1. Patient characteristics and treatment

Eleven patients (three males, eight females) aged 43 ± 16 (range 23-69) years with nephrotic syndrome entered the study and completed the protocol. All patients had a histological diagnosis of IMN from 17.0 ± 15.2 months and no evidence of diabetic glomerulosclerosis or any other secondary glomerulopathy. All patients were on full-dose ACE inhibitor (defined as the maximum dose tolerated by blood pressure) from 6 to 60 mo. (median 22 mo.), nine of them were on additional diuretics and seven on HMG CoA reductase inhibitors. The achieved daily doses of ramipril during the run-in period were 7.5 mg (n = 1), 5 mg (n = 2), 3.75 mg (n = 1), 2.5 mg (n = 4) and 1.25 mg (n = 3), respectively. The doses of ramipril, diuretics and HMG CoA reductase inhibitors were kept constant throughout the whole study period. No other RAAS inhibitors were introduced. During the treatment period, 10 subjects achieved the planned dose (100 mg/day) of spironolactone. In one patient the dose up-titration was stopped at 75 mg/day because of hyperkalemia.

### 2.2. Baseline and outcome data

**Figure 1:**
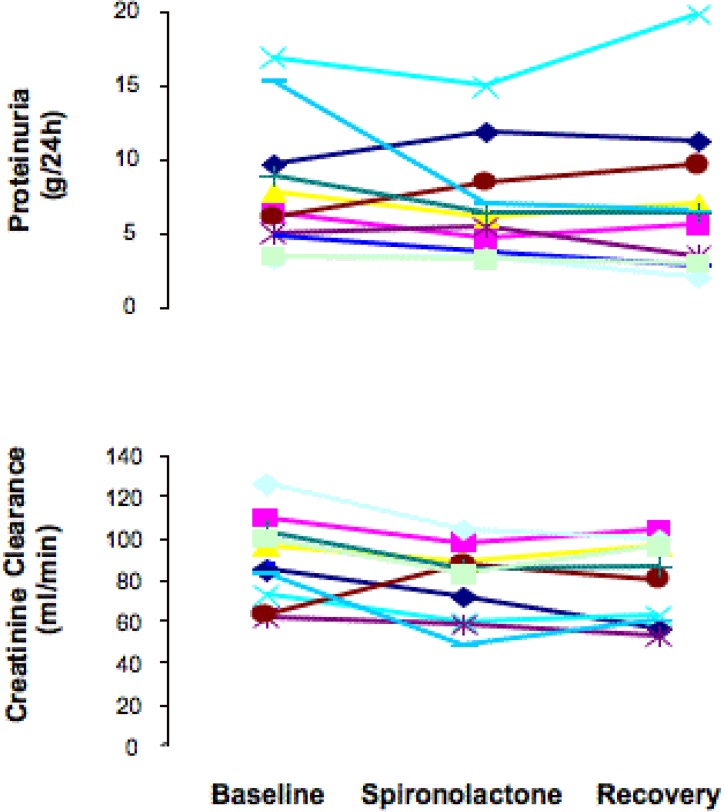
Proteinuria (upper panel) and creatinine clearance (lower panel) in each individual patient at baseline, and at the end of spironolactone treatment and of the recovery period.

**Figure 2:**
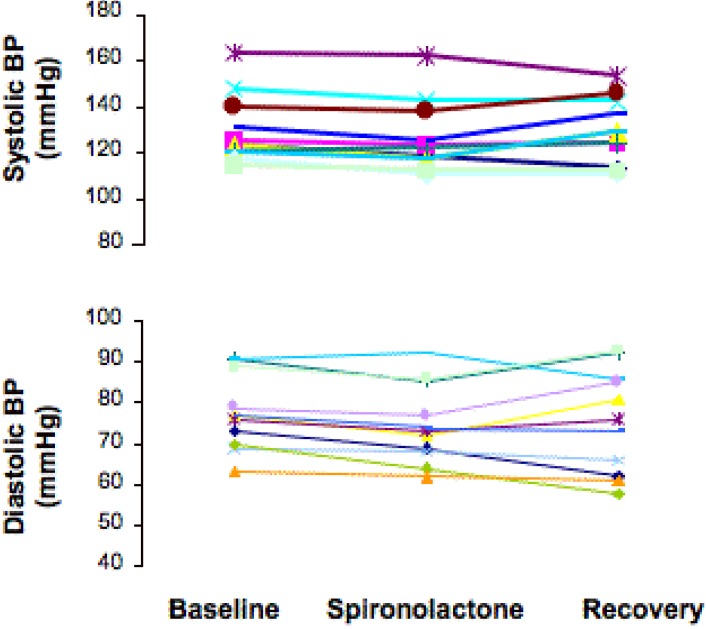
Systolic (upper panel) and diastolic (lower panel) blood pressure in each individual patient at baseline, and at the end of spironolactone treatment and of the recovery period.

At baseline evaluation, patients had heavy proteinuria and severe hypercholesterolemia (Table 1). Blood pressure was well controlled and serum potassium was in normal ranges in all patients (Table 1). Proteinuria, as quantified by either 24 h collection, or U p/c did not change between the baseline and spironolactone or recovery phase (Table 1 and Figure 1). Albuminuria did not change as well (Table 1). Both systolic and diastolic blood pressure decreased significantly after spironolactone treatment and recovered to baseline values after spironolactone withdrawal (Figure 2). Creatinine clearance followed a similar trend (Table 1 and Figure 1). Changes in proteinuria after treatment with spironolactone were positively correlated with those in creatinine clearance (-15% and –11% *vs.* baseline, respectively; r = 0.706; p = 0.013). Serum lipids did not change significantly during the treatment period. The weight had a reversible trend downward after treatment with spironolactone, but it was not significant. The mean serum potassium level significantly increased with spironolactone and recovered to baseline values after treatment withdrawal (Table 1). The increase exceeded 5.5 mEq/l in five patients and required the use of caution exchange resins in three subjects. No patient reported clinical signs or had ECG abnormalities related to hyperkalemia.

**Table 1:** Main baseline and outcome data.

	Baseline	Spironolactone	Recovery
Systolic blood pressure *(mmHg)*	129.5 ± 15	126.1 ± 15.6*	129.1 ± 14.2
Diastolic blood pressure *(mmHg)*	77.7 ± 9.27	74.7 ± 9.5*	75.7 ± 12.7
Mean arterial pressure *(mmHg)*	95.2 ± 10.6	92 ± 11.4*	94 ± 13.1
Weight *(Kg)*	69.3 ± 17.1	68.8 ± 15.5	69.7 ± 16.7
24 hour proteinuria *(g/24 hour)*	7.9 ± 4.5	6.8 ± 3.7	7.0 ± 5.1
U P/C	7.2 ± 4.8	6.5 ± 4	6.9 ± 5.2
24 hour albuminuria *(mg/24 hour)*	3790 ± 2381	3329 ± 2113	3116 ± 2289
24 hour Urinary potassium *(mEq/24 hour)*	53.8 ± 13.1	49.5 ± 16.1	51.3 ± 13.4
Urinary sodium *(mEq/24 hour)*	148 ± 32.6	159 ± 36.6	148 ± 34.3
CrCl *(ml/min)*	94.83 ± 25.7	80.32 ± 18.9**	85.19 ± 27**
Serum creatinine *(mg/dL)*	0.99 ± 0.1	1.10 ± 0.2	1.08 ± 0.2
Potassium *(mEq/L)*	4.4 ± 0.4	4.6 ± 0.4*	4.3 ± 0.3*
Serum albumin *(g/dL)*	2.27 ± 0.5	2.44 ± 0.5**	2.44 ± 0.5**
Serum protein *(g/dL*)	4.46 ± 0.6	4.77 ± 0.7**	4.74 ± 0.7**
Total cholesterol *(mg/dL)*	291.3 ± 102.5	276.2 ± 112.3	263.2 ± 80.9**
LDL-cholesterol *(mg/dL)*	201 ± 90.3	180.8 ± 63.4	184.1 ± 69.7
HDL-cholesterol *(mg/dL)*	63.1 ± 8.9	53.8 ± 10.2	50.4 ± 7.6
Triglycerides *(mg/dL)*	136.5 ± 56.2	140.5 ± 75.4	147.7 ± 56.5

Data are presented as mean ± SD; * p < 0.01, ** p < 0.05 versus baseline.

### 2.3. Discussion

In this study, add-on spironolactone therapy combined to maximally tolerated ACE inhibition failed to reduce proteinuria in patients with IMN and persistent nephrotic syndrome following treatment with ACE inhibitors alone, despite a reduction in blood pressure levels with combined therapy. Furthermore, spironolactone treatment was associated with severe hyperkalemia in three of the eleven patients that required clinical intervention with resin-binding therapy. 

In the REIN trial, higher levels of residual proteinuria after ramipril treatment were associated with poorer renal outcomes in the long run [9]. Efforts to maximally reduce proteinuria have included high dose RAAS monotherapy and the combination of ACE inhibitor and ARB up to maximally tolerated doses, which can reduce significantly urinary protein excretion and therapy improve renal outcomes in a large fraction of patients with residual proteinuria despite ACE inhibitor. 

Spironolactone add-on therapy has been proposed as an alternative strategy. Aldosterone is a mediator of fibrosis and inflammation both in the kidney and in the heart [2]. Notably, the use of ACE inhibitor alone may not achieve maximal reduction of aldosterone, and a state of aldosterone escape has been described in patients with essential [10] hypertension and diabetic nephropathy [11]. 

Studies in patients with diabetic and non diabetic nephropathies have shown a reduction in proteinuria with the combination of aldosterone antagonist and ACE inhibitor compared to ACE inhibitor alone [2,6] or ACE inhibitor with hydrochlorothiazide [7]. Chrysostomou *et al.* [6] examined the use of spironolactone (25 mg daily) in addition to enalapril in patients with CKD and persistent proteinuria. They found a 54% reduction in proteinuria after four weeks that was associated with a non significant (but clinically relevant) drop in systolic blood pressure of 8.2 mm Hg with spironolactone. A more recent trial found that in 268 subjects with type 2 diabetes and urinary albumin to creatinine excretion rate >50 mg/g, co-administration of lower doses of eplerenone (50 or 100 mg /day) with enalapril 20 mg/day reduced albuminuria to a greater extent than enalapril mono-therapy without producing clinically relevant serum potassium elevations [12]. These data have been confirmed by a recent meta-analysis, showing that, despite add-on antialdosterone therapy may reduce urinary protein excretion, this is associated with a significant risk of hyperkalemia [8]. Thus, beyond some safety concern, available clinical evidence would suggest that aldosterone blockade might represent a promising approach to reduce proteinuria and, possibly, delay progression of CKD. 

Our present results, however, invite a note of caution before considering anti-aldosterone therapy for all patients with persistent proteinuria despite ACE inhibitor therapy. Indeed, most of previous studies enrolled predominantly type 2 diabetics with nephropathy. Aldosterone escape has been described in type 2 diabetics [11], in type 1 diabetics [13] and has also been found in patients with hypertension and left ventricular hypertrophy. However, it has not been reported in non-diabetic CKD and its role in residual proteinuria in patients with membranous nephropathy is unclear. 

Differences in study design also make comparisons among trials difficult, particularly regarding baseline proteinuria levels, anti-aldosterone and ACE inhibitor doses and concomitant diuretic use. In the present study, proteinuria levels at baseline were higher that in other trials showing a beneficial effect of add-on anti-aldosterone therapy [14]. Thus, the possibility that different patient characteristics could explain different response to therapy in different studies cannot be excluded. On the other hand, insufficient aldosterone dosing unlikely explained the lack of effect on urinary proteins since in our study aldosterone doses were remarkably higher than in previous studies showing a significant proteinuria reduction [14]. Moreover, patients enrolled in this study went through an ACE inhibitor titration run in period where the ACE inhibitor was increased to the maximally tolerated (by blood pressure) dose. The other studies did not include a similar run-in period. It is unknown if the ACE inhibitor would have been more effective at higher doses in those protocols. The use of diuretics among the four studies also varies. A high salt diet may significantly impair the antiproteinuric effect of ACE inhibitors and diuretic use will restore the efficacy of the ACE inhibitor in the face of continued sodium intake [15]. This might partially explain differences between our results and those from previous studies, though compliance to low sodium diet in the present series was relatively good. A comparison of ACE inhibitor/diuretic *vs*. ACE inhibitor/aldosterone blockade may be the more appropriate comparison rather than ACE inhibitor alone *vs.* ACE inhibitor/aldosterone blockade. The decrease in creatinine clearance, increase in urinary sodium excretion and lower blood pressures during the spironolactone treatment period in the present study suggests that the diuretic effect of spironolactone may have been significant. Lastly, the number of patients in this study was small, and it is possible that the study was unable to detect a true effect. However, the negative result was still accompanied by three episodes of significant hyperkalemia.

None of the studies that showed a benefit of combined therapy with spironolactone address the long-term safety of its use in patients with CKD. In a retrospective study, Schepkens *et al.* described the development of life-threatening hyperkalemia in patients on long-term combination spironolactone/ACE inhibitor therapy, particularly in case of volume depletion. In the identified cases of hyperkalemia, combined therapy had been started on an average of 25 ± 11 weeks prior to the development of hyperkalemia. Twelve patients (out of 25) had signs of volume depletion, and nine had signs of congestive heart failure. The authors suggested that patients receiving combination therapy must be closely monitored, with particular emphasis on detecting disturbances in volume that might exacerbate the risk for hyperkalemia [16]. A recent study [17] found that following the publication of the Randomized Aldactone Evaluation Study (RALES) trial, the rate of hospitalizations and hospital deaths for hyperkalemia increased significantly. Another note of caution is that most studies have excluded patients with moderate impairment in GFR. As GFR drops, one might expect greater problems with potassium excretion and resultant hyperkalemia. Thus, the increasing enthusiasm for anti-aldosterone add-on therapy should be tempered with serious caution, since life-threatening hyperkalaemia may occur, especially at later stages of renal failure.

## 3. Experimental Section

### 3.1. Patient Selection

Patients with persistent (>6 months) nephrotic syndrome and biopsy proven IMN referred to the clinical Research Center for Rare Diseases Aldo and Cele Daccò (Bergamo, Italy) were selected for study participation. Inclusion criteria were urinary protein excretion rate persistently >3 gm/24 hours, ACE inhibitor therapy for at least 6 months, normal or moderately impaired renal function (serum creatinine <1.5 mg/dL), no previous spontaneous or treatment-associated remission, and no immunosuppressive therapy during the previous 6 months. Exclusion criteria were: Evidence of secondary membranous nephropathy, renovascular disease, obstructive uropathy, urinary tract infection, heart failure (New York Heart Association classes III-IV), atrioventricular block grades 2-3, hyperkalemia or hypokalemia (serum potassium >5.0 mmol/L or <3.5 mmol/L, respectively), stroke or acute myocardial infarction in the last three months, symptomatic coronary ischemic disease, liver or haematological disease, collagen vascular disease, cancer, chronic treatment with non steroidal anti-inflammatory drugs, known or suspected intolerance to ACE inhibitor therapy, and any condition that in the investigator’s judgment could affect study conduction or data interpretation. Pregnant, potentially child bearing or nursing women were not included as well. The Ethical Committee of the Clinical Research Center approved the study protocol and all subjects gave informed consent at entry the study, according to the Declaration of Helsinki guidelines. 

### 3.2. Treatment

This was a three-phase sequential study with a two-month run-in, a three-month treatment and a 2-month recovery phase. At study entry, patients had blood sample collections for routine laboratory analyses, an evaluation of 24-h urinary protein excretion (three timed consecutive urine collections), creatinine, urea and sodium excretion and an ultrasound evaluation to exclude urinary tract obstruction. During the two-month run-in period all patients stopped previous potassium sparing diuretics and switched from their previous ACE inhibitor therapy to ramipril starting at 1.25 mg day. The ramipril dose was then progressively increased during the first week of the run in period according to tolerability and blood pressure response (target systolic/diastolic <120/80 mmHg). The achieved dose was no more modified throughout the whole study period.

At the end of the run-in phase, patients entered a two month treatment period with spironolactone started at the dose of 25 mg once daily in the morning and increased weekly to 50 mg twice daily. The dose of concomitant diuretics was reduced in patients with symptomatic hypotension, or acute renal function deterioration (serum creatinine increase >30% versus baseline). Serum potassium was monitored within 7 days after each dose up titration and the dose was decreased to the previous level if patients developed serum potassium levels higher than 6.0 mEq/L. At the end of the treatment phase, spironolactone was stopped. The patients thereafter entered a two-month recovery phase. The ramipril was continued during the recovery phase. 

Arterial blood pressure was measured in the morning before study-drug administration by a standard sphygmomanometer and third study phase. After 5 minutes rest, three consecutive measurements were taken two minutes apart in the dominant arm with the patient in the sitting position and the mean was calculated and recorded for statistical analyses. No change in concomitant treatments was introduced through out the study period. Potassium sparing diuretics and RAAS inhibitors different from the study drugs were not allowed. A low sodium (<100 mEq/day) and a controlled protein intake (0.8 mg/kg/day) were recommended to all patients. Compliance to diet was monitored by measuring 24-hour urinary urea and sodium excretion at the end of each study period.

### 3.3. Sample size

The primary efficacy variable of the study was the 24-hour urinary protein excretion rate. Predicting (on the basis of the analysis of all IMN patients with nephrotic syndrome attending our Outpatient Clinic) a baseline urinary protein excretion rate of 8.1 ± 3.8 g/24h and assuming as clinically relevant a 60% reduction in urinary proteins, it was estimated that to give the study a 90% power to detect such reduction as statistically significant (p < 0.05) nine patients had to complete the study.

### 3.4. Statistical analysis

Data were subjected to two-way analysis of variance, and specific comparison between different mean were done using the Student’s t-test. Statistical significance is defined as P less than 0.05. Data are expressed as mean ± standard deviation (SD) or median and range, as specified.

## 4. Conclusions

The ideal use of spironolactone in the treatment of proteinuria and chronic kidney disease remains to be defined. Sequential blockade of the RAAS with ACE inhibitor and ARB has been shown to be effective in reducing proteinuria and preserving renal function in patients with non-diabetic chronic kidney disease [18]. Whether sequential blockade with ACE inhibitor and aldosterone blockade provides the same long-term benefits remains to be seen. Data from this uncontrolled study suggest that spironolactone is not helpful as an additive therapy to ACE inhibitors when patients with IMN have persistent proteinuria despite blood pressure values lower than 120/80 mm Hg, and that such treatment carries a risk of clinically significant hyperkalemia.
